# Classification and spatial characteristics of different development stages in China: Evidence from the contribution rate of production factors

**DOI:** 10.1371/journal.pone.0313069

**Published:** 2025-01-14

**Authors:** Zijie Li, Xiangnan Liu, Lingfeng Xu, Qun Wu

**Affiliations:** 1 School of Public Administration, Nanjing Agricultural University, Nanjing, China; 2 Real Estate Research Center, Nanjing Agricultural University, Nanjing, China; 3 China Institute of Resources, Environment and Development, Nanjing Agricultural University, Nanjing, China; Sapienza University of Rome, ITALY

## Abstract

This paper aims to reveal the changing characteristics of the contribution rates of different production factors in China since the reform and opening up from two dimensions: stage and space. The study used national data from 1978 to 2021 and provincial data from 2000 to 2020, combined with methods such as C-D production function and spatial econometrics for analysis. Research has found that: (1) In terms of stage characteristics, during the structural adjustment stage (1978–1998), economic growth mainly relies on capital and labor input, and the contribution rate of land factors gradually decreases. During the high-speed development stage (1998–2012), the contribution rate of technological factors gradually increased, while the contribution rate of land factors remained relatively stable. In the stage of high-quality development (2012 present), the contribution rate of technological factors continued to rise and become the dominant factor, while the contribution rate of land factors has decreased to a lower level. (2) Regarding spatial characteristics, the spatial econometric model reveals significant spatial agglomeration characteristics of capital and labor, and land factors positively affect local and neighboring economic growth. From 2000 to 2020, the contribution rate of capital factors in various provinces showed a difference of "low in the east and high in the west," which decreased year by year. The contribution rates of labor and land factors have declined to low levels in all provinces; The contribution rate of technological elements has significantly increased, with a higher contribution rate in the southeast region; High-quality development progress in each province can be identified based on the contribution rates of different production factors. The research findings help to understand the impact of varying production factors on economic development at a temporal and spatial scale and provide a scientific basis for achieving a high-quality development pattern of rational allocation of factors and regional coordinated development.

## Introduction

China’s economy has grown rapidly since the reform and opening up, with an average annual growth rate exceeding 10% for over 30 years [1]. China’s economy has transitioned from high-speed development to high-quality development [2]. In economic globalization, China faces multiple challenges, such as changes in economic growth rates, structural optimization, and power transformation, indicating the unsustainability of the traditional model based on quantitative growth. It is imperative to transition to a high-quality development model that emphasizes economic advancement’s quality and efficiency [3].

There are many studies on China’s economic development stages and high-quality development. Qi divided China’s economic development stages into primary production and industrialization stages from the perspective of the country and the city [4]. GUO revealed China’s economic inequality from 1978 to 2018 based on the center of gravity (CG) model and divided it into four development stages [5]. Wang analyzed China’s economic development model since 1978 from the perspective of environmental protection and made predictions [6]. In the face of the transformation of China’s economic development, some scholars have also analyzed the possible reasons from the perspective of socio-economics [7]. China has announced to the world that its economy is developing towards a high-quality development stage, and the academic community has also conducted research on this. First of all, the definition of connotation is mainly from the economics perspective which states that high-quality development is an economic development model, structure and dynamic system that can better meet people’s realistic and growing needs [8]. Secondly, most of the research is on the measurement of high-quality development [9], and there are also measurements of high-quality economic development for industries [10]. Still, there is no unified measurement system to measure China’s high-quality economic development. Existing studies have proven that factors such as the digital economy [11], environmental regulation [12], and industrial agglomeration [13] have a significant impact on high-quality development. However, the reason is that the high-quality development of China’s economy is inseparable from the rapid advancement of scientific and technological innovation and economic globalization. With the United Nations proposing the SDGs [14], China is meeting this challenge with the transformation of its development model and has achieved remarkable results. In summary, although there are many studies on China’s economic development stage and high-quality development, few consider these issues from the perspective of the contribution rate of production factors.

China is the world’s largest energy consumer, with coal consumption nearly quintupling between 1978 and 2017. Additionally, arable land continuously decreased since the 21st century [15]. These excessive resource consumption patterns have led to environmental degradation, necessitating reevaluating the economic development model [16]. China’s economy has historically relied heavily on capital and labor, leading to an extensive growth model characterized by high energy consumption, pollution, and input costs [17]. This resource-depleting growth model is unsustainable and no longer suitable for the new economic normal, nor does it align with the current international environment. This economic growth model, which comes at the cost of continuous resource depletion, is bound to be unsustainable, and the limits of scale expansion and economic growth are no longer in line with the current global sustainable development goals [18]. Therefore, it is necessary to abandon the previous model of quantity-driven extensive growth and transition to sustainable development [19]. China’s existing resource and environmental conditions have seriously constrained sustained economic growth [20]. Research shows that with economic development, scientific and technological innovation and optimization and upgrading of industrial structure have become the main driving forces for China’s economic development today [21]. The Chinese government realizes that the marketization of factors is an important direction for promoting high-quality economic development [22]. Promoting the orderly flow and optimal allocation of resources and factors among regions is of great significance [23] for effectively stimulating the potential of various production factors and unblocking the international economic cycle.

In summary, existing research has only studied the stage division and the changes in the contribution of different production factors, and there has not yet been a division of development stages based on the perspective of the contribution rate of production factors. The main objective of this study is to comprehensively explore the characteristics of other factors’ contribution rates from the perspectives of economic development stages and provincial spatial distribution. Firstly, by examining the inflection points of different factors’ contribution rates over the years, this study will identify the various stages of China’s development and analyze their changing characteristics. Secondly, the spatial attributes of production factors and their contribution rates across all provinces in China since 2000 will be examined. Finally, further policy suggestions are put forward for the main conclusions of the research, which can provide a basis for other countries to understand China’s economic development strategies better, and offer a new perspective for global economic theory and empirical research.

## Methods and data sources

### Research methods

#### (1) C-D production function

In economic theory, the contributions and differences of different factors in economic development can be reflected through production functions [24]. A production function is a function that describes the relationship between factors in the production process, and the Cobb-Douglas production function is a representative tool for measuring factor contribution rates. It can not only calculate the contribution rate of capital and labor but also expand to increase other factors of production [25], so it has been widely used [26,27]. Therefore, in this study, the Cobb-Douglas production function is selected to calculate the contributions of various factors of production to economic growth. The production functions considered in this research include capital, labor, land, and technology. Therefore, the expression of the production function is as follows:

$$Y=Ae^{rt}K^{\alpha_{1}}L^{\alpha_{2}}S^{\alpha_{3}}\mu$$ (1)

Where *Y* is the level of economic growth, *A* is a constant greater than 0, representing the initial level of technology, *K*、*L*、*S* represent capital input, labor level, and land input, respectively, *t* represents the time variable, *α*_1_、*α*_2_、*α*_3_ represent the output elasticity coefficients of capital, labor, Taking the logarithm of both sides of Eq (1):

$$LnY=LnA+rt+\alpha_{1}LnK+\alpha_{2}LnL+\alpha_{3}LnS$$ (2)

Based on Eq (2) and utilizing historical time-series data, least squares regression method is employed to calculate the corresponding values of the production factors *α*_1_、*α*_2_、*α*_3_. This enables the determination of the contribution rates of each production factor to economic growth.

The contribution rate of technology (A) to economic growth is calculated as follows: $E_{r}=\frac{r}{\Delta Y}$, the contribution rate of capital (K) to economic growth is calculated as: $E_{k}=\alpha_{1}\frac{\Delta K}{\Delta Y}$, the contribution rate of labor (L) to economic growth is calculated as: $E_{l}=\alpha_{2}\frac{\Delta L}{\Delta Y}$, and the contribution rate of land (S) to economic growth is calculated as: $E_{s}=\alpha_{3}\frac{\Delta S}{\Delta Y}$.

#### (2) Spatial econometric model

Scholars often employ spatial econometric models for measurement [28] to investigate the spatial characteristics of various production factors, with the Spatial Durbin Model (SDM) being widely used. In addition to the spatial impact of its own growth, economic growth cannot ignore the influence generated by the spillover effects of other potential factors, such as the spatial effects of input factors. The construction of the model through the SDM model provides unbiased parameter estimation results, effectively addressing the issues of spatial lag errors and the inability to identify parameters caused by spatial lag. Based on this, a general spatial econometric model is constructed as follows:

$$\left\{ \begin{array}{l} Y_{it}=\rho \sum_{i,j=1}^{31}W_{ij}Y_{it}+\beta X_{it}+\theta \sum_{i,j=1}^{31}W_{ij}X_{it}+\mu_{i}+\nu_{i}+\varepsilon_{it}\\ \varepsilon_{it}=\lambda \sum_{i,j=1}^{31}W_{ij}\varepsilon_{it}+\varphi \end{array}\right.$$ (3)

Where *Y*_*it*_ is economic growth indicator for each province-year, *X*_*it*_ is explanatory variables (capital, labor, technology, and land), *ρ* and *λ* represent the spatial autocorrelation coefficients, *β* and *θ* represent the coefficients of the explanatory variables and spatial spillover, *W*_*it*_ is adjacency spatial weight matrix, indicating the spatial association between provinces *i* and *j*, *u*_*i*_ and *v*_*i*_ represent the spatial effects and time effects, respectively, *ε*_*it*_ is a random error term, *φ* is the random error term following a normal distribution.

### Data sources

This study utilizes cross-sectional data from 1978 to 2021 in China and panel data spanning 21 years (2000–2020) from 31 provinces (municipalities directly under the central government) for estimation. All statistical data are sourced from the ’China Statistical Yearbook 1978–2022’, ’China Urban Statistical Yearbook 2000–2021’, ’China Urban Construction Statistical Yearbook 2000–2021’, as well as the statistical yearbooks of respective provinces (municipalities directly under the central government) for each year. The vector map data is obtained from the National Geographic Information Resource System’s publicly available 1:1,000,000 primary geographic database(http://www.webmap.cn). The spatial coordinate system used is the China Geodetic Coordinate System 2000, based on the national geodetic coordinate system as of 2000. The detailed explanation of different production factors is shown in Table 1.

**Table 1:** Description of various production factors.

Elements	Symbol	Meanings	Unit	Instruction
Economic Growth	Y	Value-added value of the secondary and tertiary industries	100 million	Use the GDP deflator to eliminate the impact of price changes
Capital	K	Stock of capital	100 million	Using the perpetual inventory method [29]
Labor	L	Population employed in the secondary and tertiary industries	thousands of people	-
Land	S	Area of urban construction land	Km^2^	-

## Contribution rates of different production factors: Stage characteristics

### Ridge regression analysis

**Fig 1:**
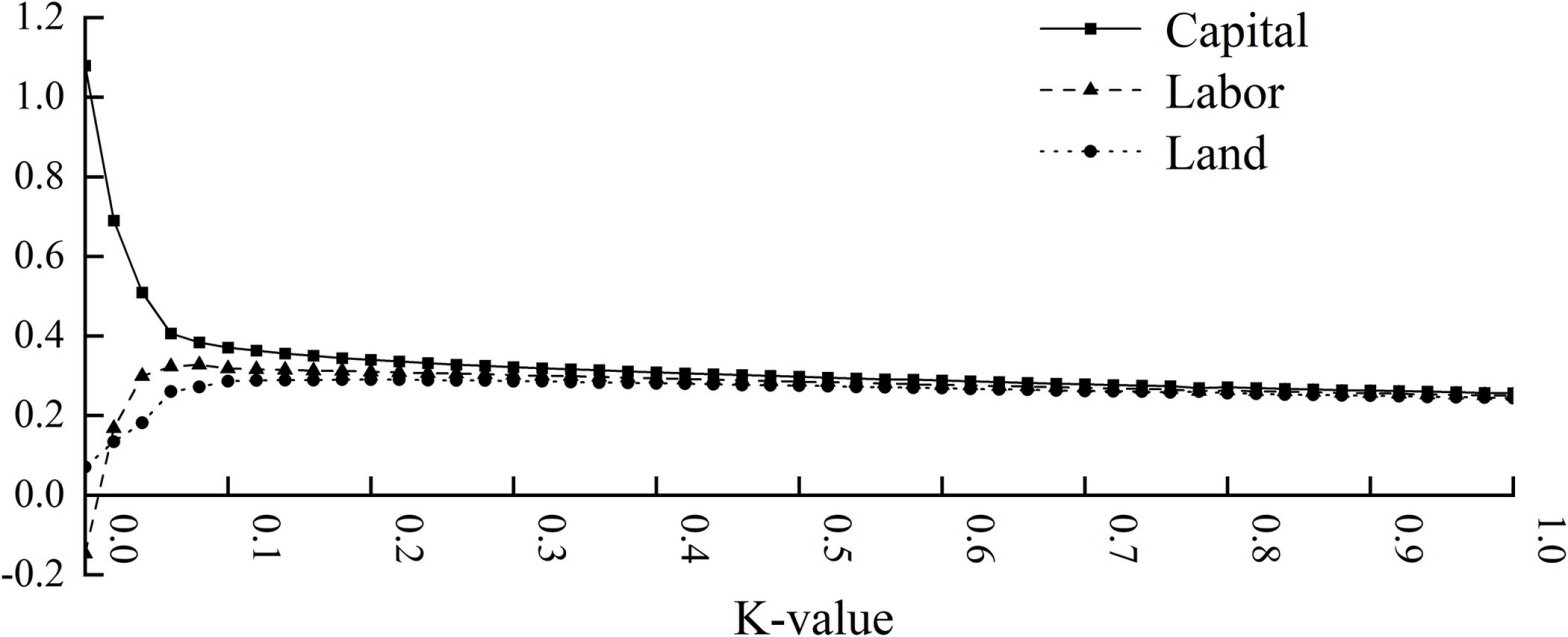
Production function regression ridge trace.

Ridge regression analysis [30] was conducted on the data of various production factors from 1978 to 2021, as indicated in Eq (2), using SPSS 27.0 statistical analysis software. As shown in Fig 1, the regression coefficients of different production factors exhibit rapid changes with increasing values of k(Table 2). Specifically, the regression coefficient of capital factors decreases significantly, while the regression coefficients of labor and land factors increase rapidly. After adjustment, it was found that when R^2^ = 0.9840 and k = 0.34, the overall regression coefficients tend to stabilize. It can be observed that the regression coefficient for the capital factor is 0.316, the labor factor is 0.299, and the land factor is 0.284, all of which have passed the significance test at the 1% level.

**Table 2:** Regression coefficients of production function.

Index	Regression coefficients	Standard error	VIF
*LnK*	0.316^***^	0.04	2.986
*LnL*	0.299^***^	0.095	4.401
*LnS*	0.284^***^	0.174	4.964
*Constants*	4.022	0.045	
*Ridge regression parameters*		0.36	
*Adjusted R* ^*2*^		0.983	

^*^ *p < 0*.*05*, ^**^ *p < 0*.*01*,

^***^ *p < 0*.*001*.

### Temporal changes in contribution rates of different production factors

By calculating and analyzing the contribution rates of production factors over the past 40 years of reform and opening up, the contribution rates of production factors in different years are shown in Table 3.

**Table 3:** Contribution rates of various production factors from 1980 to 2021.

Year	Capital	Labor	Land	Technology
1980	26.98%	35.46%	30.15%	7.41%
1985	27.02%	33.94%	30.05%	8.99%
1990	26.78%	32.38%	27.65%	13.20%
1992	26.89%	29.04%	25.57%	18.50%
1995	27.41%	26.05%	24.28%	22.26%
1998	27.61%	25.28%	22.69%	24.42%
2000	27.39%	24.57%	22.31%	25.73%
2002	28.28%	24.37%	24.23%	23.13%
2005	29.01%	23.42%	21.82%	25.75%
2007	28.81%	22.44%	21.32%	27.44%
2010	29.38%	21.42%	20.34%	28.86%
2012	29.99%	21.40%	20.59%	28.03%
2015	30.26%	20.96%	20.25%	28.53%
2017	30.05%	18.79%	18.05%	33.12%
2019	29.53%	20.01%	19.59%	30.87%
2020	29.88%	20.22%	19.76%	30.14%
2021	29.21%	19.67%	19.42%	31.71%

The contribution rate of capital factors has slightly declined after a continuous rise. It has been increasing since 1980 and reached 30.26% in 2015, then began to decline and fell to 29.21% in 2021. The trend of capital factor is closely related to the transformation and adjustment of China’s economic structure [31]. In the early days of reform and opening up, capital investment was the main driving force for economic growth, consistent with the role of capital accumulation in economic growth described by the Harrod-Domar model [32]. However, as the economy underwent a transformation and structural adjustment, the marginal contribution of the capital factors gradually weakened. In the stage of high-quality development, further optimization of economic and industrial structures has led to a continuous decline in the contribution rate of capital factor.

The contribution rate of labor factor has continuously declined, from 35.46% in 1980 to 19.67% in 2021, a decrease of nearly 45%. This change is primarily due to changes in population structure and the supply-demand relationship in the labor market under different economic development models. Lewis’s dual economy model can explain this. As China’s demographic dividend gradually fades, the population shifts from agriculture to industry and services, and the supply pressure in the labor market increases, resulting in a downward trend in the contribution rate of labor factors [33]. Simultaneously, as we enter a stage of high-quality development, our scientific and technological levels are increasingly improving, and technological innovation-driven development is taking a dominant position [34], further weakening the contribution of labor factors to economic growth.

The contribution rate of technological factors have significantly increased, rising from 7.41% in 1980 to 31.71% in 2021, becoming the most significant contributing production factor. Endogenous growth theory believes that technological progress plays a crucial role in economic growth [35], closely related to the Chinese government’s policy of increasing innovation and promoting scientific and technological progress. China has made significant scientific and technological progress by increasing investment in R&D and cultivating innovative enterprises. The rising contribution rate of technological factors reflects China’s important position in the new round of global scientific and technological revolution and industrial revolution and its remarkable achievements in the development of high-tech industries and a knowledge economy [36].

The contribution rate of land factor has shown a fluctuating downward trend since China’s reform and opening up. It has decreased from 30.15% in 1980 to 19.42% in 2021. This downward trend is related to the substitutability of factors. Various factors, such as industrial structure upgrading, technological progress, and management optimization, have replaced the input of traditional capital and labor factors. As the economy transitions from a land-intensive extensive mode to a mode focused on technological and institutional optimization, the contribution rate of land factor continues to decline. The temporary increase in the middle reflects the increased demand for land development and utilization during certain stages. Still, the subsequent decline may be influenced by resource and environmental constraints and the compression of land development space, leading to further substitution of other factors for land [37].

Under the background of high-quality development, through in-depth analysis, it is found that the contribution rates of various production factors have exhibited significant changes from the reform and opening up to the stage of high-quality development. Capital factors played a dominant role in the early stages. In contrast, technological factors gradually became dominant with the arrival of economic transformation and the stage of high-quality development. The contribution rates of labor and land factors continued to decline. The contribution rates of production factors are closely related to the stage of economic development. Different stages of economic development exhibit different characteristics in terms of factor contribution rates. Therefore, further research on the stage characteristics of production factors is needed to provide references for regions at various stages of development and future development.

### Analysis of the characteristics of factor contribution rates in different stages

**Fig 2:**
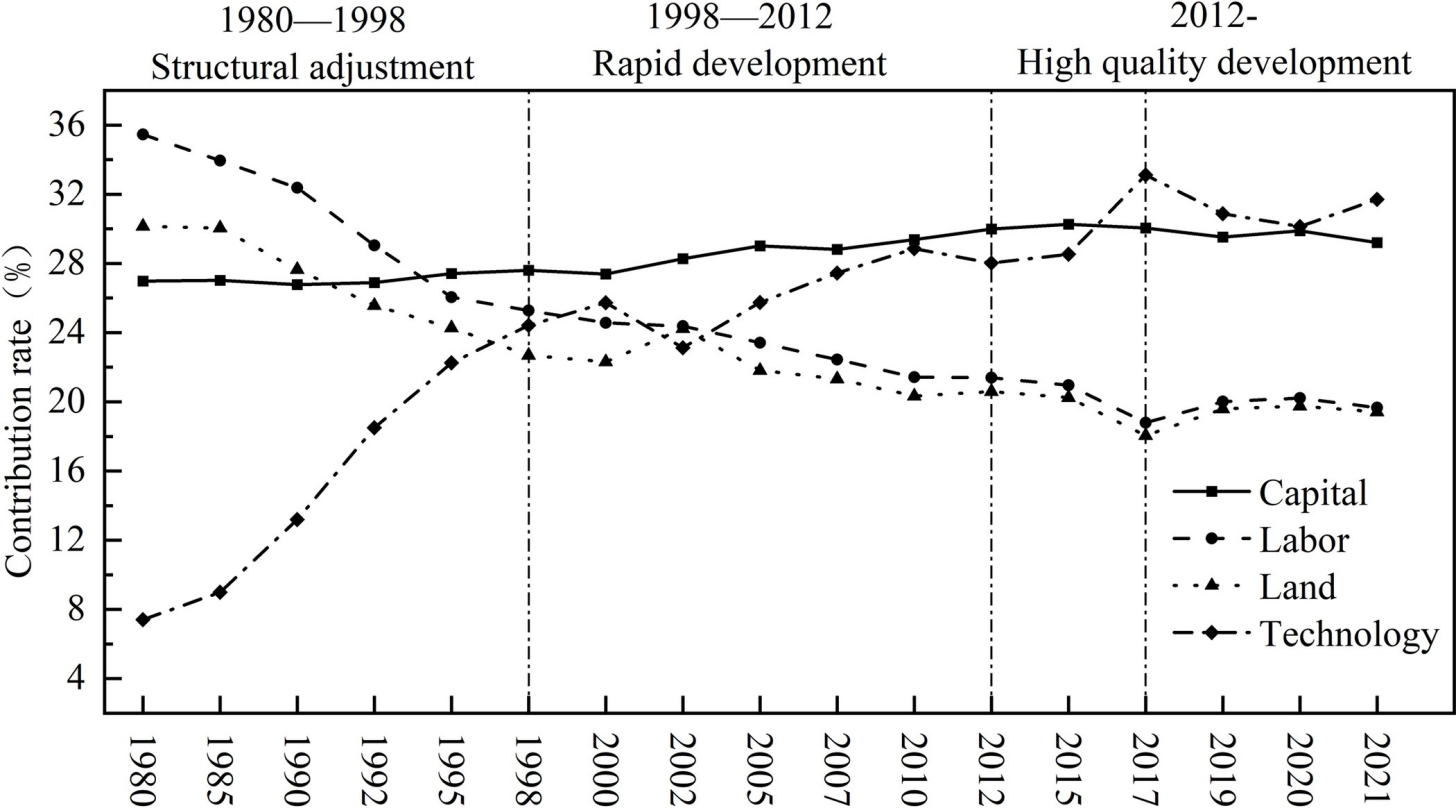
Contribution rates of various production factors at different development stages.

Economic development occurs in stages, and at different stages, the economic development relies on different resource endowments, objectives, and tasks, thereby exhibiting different development characteristics [38]. In China, different policy orientations and focuses are employed in various stages of economic development, and the indicator characteristics of production factors also exhibit significant differences [39]. Fig 2 shows the results of the stages division.

The structural adjustment stage (1978–1998) began China’s economic development. In the early stages of the reform and opening-up, inadequate consideration of the actual economic growth led to overheating of the economy [40]. In 1977, the Chinese government launched many construction projects, which resulted in increased resource consumption and damage to the ecological environment. In 1992, to promote the reform of establishing a socialist market economic system, the government took various measures to stabilize the economy. At this stage, the contribution rate of different production factors fluctuated wildly with the adjustment of the economic structure. China’s rapid economic growth since 1980 has mainly relied on the injection of large amounts of capital and labor, and it is still an extensive investment-based growth. At this stage, the contribution rate of capital factors stabilized at more than 26% and showed an increasing trend; the contribution rate of labor factors dropped significantly after 1995, with a decline rate of more than 28%; as a representative of resource consumption, the contribution rate of land factors continued to decline. This is because China’s land system began to change in the 1980s truly, and urban land use rights can be transferred according to law [41]. The government is committed to releasing the demographic dividend and accelerating the land capitalization. At this stage, various construction projects are in full swing, resulting in a significant increase in the contribution rate of technical factors. The characteristics of this stage were rapid economic growth but a lack of balance, mainly relying on factor inputs and exposing resource consumption and environmental issues. The problems brought about by rapid economic growth prompted the Chinese government to adjust and transform the financial structure promptly, laying the foundation for transitioning to a stage of high-quality development.

The high-speed development stage (1998–2012) was an essential stage of China’s economic development. During this period, the overall living standards of the Chinese people have improved significantly. Economic growth has led to the rapid expansion of production capacity. The contradiction of insufficient domestic effective demand has gradually become apparent, and the economic growth rate has slowed. At the same time, affected by the impact of the international financial environment, to avoid continued economic decline, the country implemented several economic stimulus plans [42]. At this stage, the contribution rates of various production factors changed significantly, capital factors played the main contribution at this stage, and the contribution rate continued to rise to 29.99%, the contribution rate of labor factors continued to decline to 21.4%. At this stage, population mobility is accelerating, and the labor market is in short supply. The government needs to optimize the land use structure through land policies, improve land use efficiency, and promote economic growth. A series of government land marketization policies have accelerated the improvement of land mobility and utilization efficiency [43]. At the same time, the urbanization process has accelerated, and the contribution of land factors has temporarily increased. In 2010, the Chinese government began implementing real estate market control policies, making the overall land contribution rate relatively stable [44]. The contribution rate of technical factors continued to rise after declining in 2002, reaching 28.86% in 2010, almost the same as the contribution rate of capital factors, occupying an important position among various production factors. At this stage, China’s economic growth was stable. However, with the advancement of market-oriented policies, constraints on factors such as land and labor have intensified, making technological innovation more difficult. This change shows that the Chinese government’s economic policies significantly impact the contribution rate of production factors, which also verifies that institutional economics believes that economic growth is affected by the institutional and policy environment. The government’s adequate institutional arrangements and policy framework can improve market efficiency and increase the allocation efficiency of production factors [45].

The high-quality development stage (2012 to present) is a new stage of China’s economic development. During this stage, the overall macro-economy faced the problem of structural imbalance. In 2017, the government proposed accelerating the transformation of the economic development mode and shifting the focus of development to improving quality and efficiency, marking the Chinese economy’s move towards a high-quality development stage [46]. Therefore, the contribution rate characteristics of various production factors in this stage can be divided into two sub-stages, with 2017 as the boundary:

2012–2017, the initial construction stage of high-quality development. In 2012, the government optimized the allocation of land resources with a series of policies. It proposed to provide sufficient land to the market according to the annual plan to meet market demand. The interactive relationship between land factors and capital, labor, and other factors changed accordingly. The contribution rate of capital factors in this stage was generally stable at around 30%, and the contribution rate of labor factors dropped from 21.40% to 18.79%, reaching the lowest level. The contribution rate of land factors and the contribution rate of labor factors dropped by the same amount. It is worth noting that the contribution rate of technical factors has increased significantly. In 2017, it exceeded the contribution rate of capital factors for the first time, reaching 33.12%. This is also the manifestation of the characteristics of high-quality development, and it has gradually become an active driving force for economic development.

Since 2017, China has entered a stage of high-quality development. Scientific and technological innovation has become the first driving force leading China’s economic growth, meeting the viewpoint of knowledge economy theory [47]. The contribution rate of production factors in this stage is reflected in the following aspects: the contribution rate of technical factors reached 31.71%, which dominated all kinds of production factors; the contribution rate of capital factors dropped to 29.21%, but still maintained a high level; the contribution rate of labor factors continued to decline, and the contribution rate of land factors showed the same trend of change. This phenomenon is attributed to the continuous compression of the incremental development space of land resources, the steady improvement of the efficiency of stock land use, and the gradual shift of land resources from quantitative contribution to quality and efficiency improvement. This series of trends shows that China’s economy is gradually shifting from resource-dependent growth to high-quality, growth relying on technological innovation and factor allocation optimization, which aligns with China’s high-quality goals. China’s economy has achieved a nearly perfect transformation by optimizing resource allocation, improving the level of technological innovation, and improving the institutional environment.

## Contribution rates of different production factors: Spatial characteristics

The differences in economic development levels and resource endowments among regions can lead to significant variations in the contribution rates of production factors across different areas. Spatial econometric analysis can reveal the spatial correlations between different regions and provide a deeper understanding of the role of production factors in regional economic growth [48]. Therefore, the data from 31 provinces in China from 2000 to 2020 were further analyzed using spatial econometrics methods (To delve into the analysis of the high-quality development stage, we only discuss the situation after 2000, and data from Hong Kong, Macau, and Taiwan were excluded.).

### Model verification and identification

**Fig 3:**
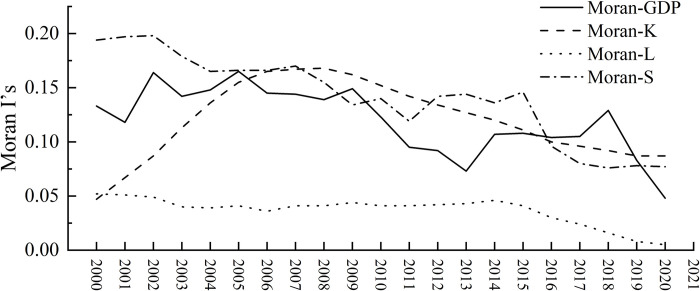
Time series chart of GDP and Moran’s I index of production factors from 2000 to 2020.

Before estimating the spatial econometric model, it is necessary to test the spatial correlation between the production factors and economic growth among the 31 provinces using Moran’s I index, which measures the degree of spatial association between them [49]. As shown in Fig 3, the Moran’s I index for GDP ranged from 0.048 to 0.165 from 2000 to 2020. The Moran’s I index for capital factors ranged from 0.047 to 0.168; for labor factors, it ranged from 0.005 to 0.052; and for land factors, it ranged from 0.076 to 0.198. Moreover, all three production factors passed the 1% significance level, indicating significant spatial autocorrelation between interprovincial input of production factors and economic growth in China.

When selecting the specific form of the spatial panel econometric model, it is necessary to examine the manifestation of spatial dependence and then determine whether the Spatial Durbin Model (SDM) can be simplified into Spatial Lag Model (SLM) and Spatial Error Model (SEM) using the Wald test and LM test. Initially, an ordinary least squares (OLS) regression model was applied to establish the ordinary regression model, and there were no issues of multicollinearity (VIF < 10) [50]. The OLS test results indicated that the LM test statistics of the spatial lag model were significant at the 1% level, rejecting the null hypothesis of no spatial lag. The LM test for the spatial error model also showed that the R-LMerr was significant at the 1% level. The Hausman test results indicated the selection of the fixed effects model. Based on this, the Spatial Durbin Model (SDM) was constructed, and further Wald and LM tests demonstrated that the SDM could not be simplified into SLM and SEM. In conclusion, this study selected the Spatial Durbin Fixed Effects Model (Table 4).

**Table 4:** Model test results.

LM test	T	P
*LMerr*	80.304	0.000^***^
*R-LMerr*	27.922	0.000^***^
*LMlag*	94.856	0.000^***^
*R-LMlag*	42.474	0.000^***^
*Hausman test*	46.97	0.000^***^
*Moran’s I*	9.477	0.000^***^

^*^ *p < 0*.*05*, ^**^ *p < 0*.*01*,

^***^ *p < 0*.*001*.

### Estimation results of panel data

Firstly, the traditional OLS regression method was employed, revealing that capital, labor, and land all positively affect economic development, with land having the slightest impact. The previous Moran’s I values indicated significant spatial autocorrelation between various production factors and economic growth. Since the traditional OLS model cannot capture spatial effects, it is necessary to introduce spatial econometric models for estimation.

Using Stata software, a regression analysis of the panel data was conducted for three spatial econometric models (Table 5). In terms of the goodness of fit measured by the R-squared (R^2^), the Spatial Durbin Model (SDM) exhibited the highest goodness of fit (0.896), providing more accurate estimation results compared to the other two models. The spatial correlation coefficients passed the 1% significance test, indicating significant spatial spillover effects in regional economic growth and further validating the need to incorporate spatial effects into the model. Comparing the log-likelihood values (Log-L), the SDM model (-25.949) was significantly higher than the SLM model (-39.913) and SEM model (-37.026). Further analysis of the estimation results of the SDM model is as follows.

1. Regarding regression coefficients, capital, labor, and land factors were estimated to be 0.350, -0.011, and 0.416, respectively, and all passed the 1% significance test. This indicates that a 1% increase in capital and labor factors leads to a 0.35% and 0.416% increase in economic development, respectively. The negative coefficient for labor suggests that it does not positively impact economic growth.
2. Regarding the spatial correlation coefficients, the spatial lag model (SLM) had the highest coefficient (0.288) since the inclusion of spatial lag terms increases the impact. The spatial error model (SEM) had the lowest coefficient (0.157), while the Spatial Durbin Model (SDM) had a coefficient (0.222) between the two.
3. Analyzing the spatial lag parameters of the explanatory variables, the coefficients for W-K, W-L, and W-S were -1.920, -0.428, and 1.839, respectively. The estimation results indicate that while land factors have a positive promoting effect, factors such as capital and labor, which are more mobile, negatively affect neighboring provinces’ economic growth. The economic growth of provinces involves both agglomeration and diffusion effects of factors. According to the estimation results, the ability of interprovincial capital and labor to agglomerate is significantly higher than their diffusion ability in China. As provinces experience economic growth, economically developed provinces attract capital and labor from surrounding provinces, causing their concentration and continuous outflow of these factors to affect the surrounding provinces negatively. This is because in the high-quality development stage driven by technological innovation, economic and industrial development is more focused on innovation and knowledge-intensive industries, which have a relatively lower demand for capital and labor but a higher demand for land factors. As a limited and immobile resource, land factors promote the economic growth of their respective regions. They can influence the economic growth of adjacent regions through spatial spillover effects. To achieve high-quality economic development, it is essential to effectively improve factor allocation efficiency, particularly for mobile factors such as capital and labor. In-depth exploration is needed to balance regional allocation and optimize the quality, mode, and structure of factor allocation, thereby enhancing economic growth dynamics, quality, as well as efficiency and promoting high-quality economic development.

**Table 5:** Spatial econometric model estimation results.

Variable	OLS	SLM	SEM	SDM
*K*	0.608^***^	0.356^***^	0.351^***^	0.350^***^
	(0.00)	(0.00)	(0.00)	(0.00)
*L*	0.553^***^	0.050^***^	0.046^***^	-0.011^***^
	(0.00)	(0.57)	(0.60)	(0.90)
*S*	0.029^***^	0.391^***^	0.398^***^	0.416^***^
	(0.50)	(0.00)	(0.00)	(0.00)
*W-K*				-1.920^***^
				(0.00)
*W-L*				-0.428^***^
				(0.54)
*W-S*				1.839^***^
				(0.00)
*rho*		0.228^***^	0.157^***^	0.222^***^
		(0.00)	-0.03	(0.02)
*Observations*	651	651	651	651
*Log_L*		-39.913	-37.026	-25.949
*Adjusted R* ^*2*^	0.920	0.876	0.886	0.896

^*^ *p < 0*.*05*, ^**^ *p < 0*.*01*,

^***^ *p < 0*.*001*.

### Spatial analysis of provincial production factors’ contribution rate

**Fig 4:**
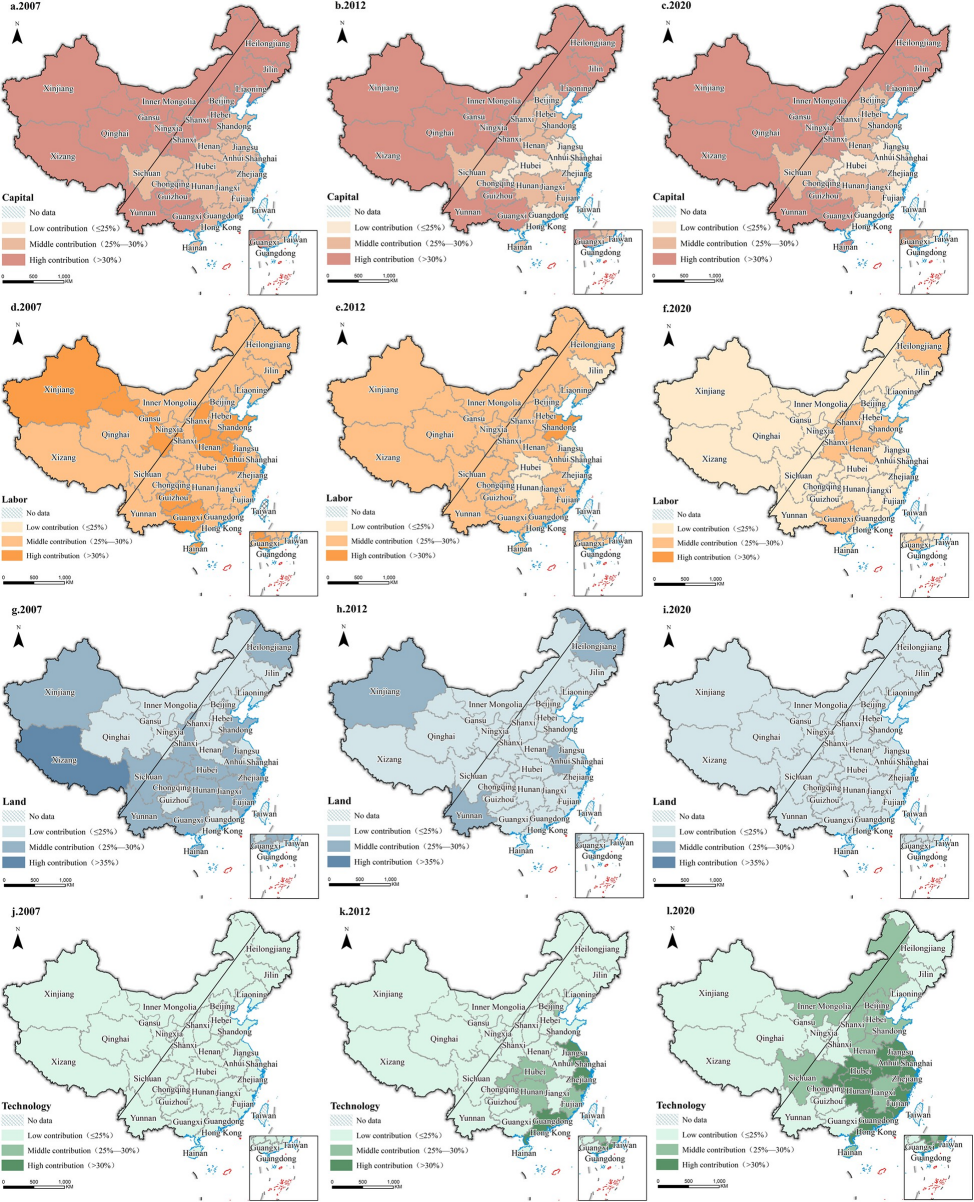
Distribution of contribution rates of production factors in each province from 2000 to 2020.

The contribution rates of production factors for each province in China were calculated and analyzed using the same method, as shown in Fig 4 and the appendix. The results indicate significant changes in the contribution rates of various production factors, which generally follow the national trend.

Capital factors exhibit a clear east-to-west regional trend. From 2000 to 2020, the contribution rates of capital in the eastern three provinces and western regions, such as Guizhou, Yunnan, Tibet, Qinghai, Ningxia, and Gansu, showed an increasing trend. This is due to the strategic support of the national Western Development policy and the internal demand for economic development in the Western provinces, leading to a high contribution rate of capital in these regions [51] Coastal provinces in the eastern and Yangtze River Economic Belt experienced a significant decrease in their contribution rates of capital after entering the stage of high-quality development in 2012. This is attributed to their regional and policy advantages, which attracted a large influx of capital and reduced their reliance on capital factors, significantly dropping contribution rates to a low level (≤25%).

Labor factors experienced a significant decrease. In 2007, regions with high contributions (>35%) from labor factors were concentrated in northern provinces like Shanxi, Henan, and Shandong. However, after entering the high-quality development stage in 2012, factors such as the increasing level of mechanization and the shift from labor-intensive industries to technology-intensive and capital-intensive industries [52] led most provinces to decrease their contribution rates to a low level (≤25%). Due to their large population base, central provinces still rely on labor factors to some extent, but their contribution rates have also decreased to a moderate level (25%–30%).

The contribution rate of land factors has been decreasing year by year. With the acceleration of urbanization and industrialization after China’s reform and opening up, it can be observed that in 2007, southern regions had a high dependence on land, with most of them at a moderate contribution level (25%–30%) or above. By 2020, as land resources became scarcer and more valuable, land utilization efficiency improved, and national regulations strengthened, provinces across the country primarily reduced their reliance on the number of land factors and shifted their focus toward land quality and efficiency [53]. Developed regions such as Beijing, Shanghai, Jiangsu, and Zhejiang now have contribution rates below 20%.

Technological factors represent the most significant production factor in terms of high-quality development. In 2007, the contribution rates of technological factors were generally low (≤25%) nationwide because, at that stage, economic growth still relied on low-cost labor and high resource inputs, and technology was in its infancy in terms of development and application. After 2012, with the upgrading and transformation of industrial structure and the shift from "factor-driven" to "innovation-driven" development [54], the contribution rates of technological factors in developed coastal cities in the eastern regions significantly increased. By 2020, with the continuous improvement of national technological innovation capabilities and technology transfer, the contribution rates of technological factors in various provinces showed significant improvements. Due to their inherent resource advantages, most eastern provinces have medium to high contribution rates, especially the developed coastal regions with high contribution levels (>30%). In contrast, the contribution rates of technological factors in economically lagging regions in central and western China and the northeast remain relatively low.

Reprinted from the National Geographic Information Resources Directory Service System(http://www.webmap.cn)

### Regional division of high-quality development

The problem of unbalanced regional economic development in China is one of the biggest challenges faced in high-quality development. China attaches great importance to the regional differentiation caused by the widening gap in regional economic development and regional economic layout [55]. Under such a development background, China’s regional development pattern will undergo specific changes. Due to various reasons, the contribution rate of production factors to economic development in multiple provinces across the country has changed to a certain extent. Chinese scholars have paid attention to the problem of unbalanced economic development in China [56] and have conducted research on China’s regional division, including from a geographical perspective based on landforms [57], reclassification of small regional areas of national main functional regions [58] and regional division based on carbon intensity [59]. Li’s research focused on the regional differences brought about by high-quality development [60], but current research has not yet focused on the progress of high-quality development in various regions. To further grasp the progress of each province in high-quality development, this part of the study summarizes the changes in the contribution rate of production factors. It proposes corresponding division standards to determine the progress of each province in entering the stage of high-quality development and provide a specific basis for regional coordinated development:

1. When the contribution rates of capital, labor, and land factors are all below 20%, and the contribution rate of technological factors is not lower than 40%, it can be classified as having entered the high-quality development stage.
2. When the contribution rate of capital factors is not less than 35%, and the contribution rates of labor and land factors are above 20%. In comparison, the contribution rate of technological factors is less than 20%. It is classified as not having entered the high-quality development stage.
3. In other cases, it is classified as being in the quasi-high-quality development stage.

Table 6 shows division results, only four provinces (municipalities)—Beijing, Shanghai, Jiangsu, and Zhejiang—have entered the high-quality development stage. Meanwhile, regions in the western part of the country, such as Guangxi, Ningxia, Xinjiang, Tibet, Qinghai, Guizhou, Yunnan, and Gansu, have not yet entered the high-quality development stage. The remaining provinces are classified as being in the quasi-high-quality development stage. This division criteria’s scientific and accurate nature requires further research and verification. Still, it provides a preliminary framework better to understand the progress of each province in high-quality development, serving as a reference for further achieving high-quality regional development.

**Table 6:** High-quality development process of each province.

Type	Province	Criteria for the classification
Has entered into high-quality development	Beijing, Shanghai, Jiangsu, Zhejiang	Capital factors ≤ 20%; labor factors ≤ 20%; land factors ≤ 20%; technical factors ≥ 40%
On the verge of entering high-quality development	Tianjin, Chongqing, Hebei, Shanxi, Inner Mongolia, Liaoning, Jilin, Heilongjiang, Anhui, Fujian, Jiangxi, Shandong, Henan, Hubei, Hunan, Guangdong, Hainan, Sichuan, Shaanxi	Other cases
Not yet entering high-quality development	Guangxi, Ningxia, Xinjiang, Tibet, Qinghai, Guizhou, Yunnan, Gansu	Capital factor ≥ 35%; labor factor > 20%; land factor > 20%; technical factor < 20%

## Conclusion and recommendations

### Conclusions

Under the current context of China’s transition to high-quality development, this study conducts a comparative analysis of the contribution rates of production factors to economic growth during different stages from 1978 to 2021. Additionally, it analyzes the spatial characteristics of these factors using provincial data from 2000 to 2020. The following conclusions are drawn:

1. The contribution rates of production factors exhibit distinct stage-specific characteristics during different stages of economic development. During the structural adjustment stage (1978–1998), China’s rapid economic growth relied mainly on the input of capital and labor factors, while the contribution rate of the land factor showed a declining trend. In the high-speed development stage (1998–2012), the new driving force behind economic growth was the technology factor, with its contribution rate gradually increasing while the contribution rate of the land factor remained relatively stable. In the high-quality development stage (2012-present), the technology factor became dominant, with its contribution rate increasing yearly. In contrast, the contribution rate of the land factor declined to a lower level.
2. The spatial characteristics of inter-provincial production factors are revealed by constructing a spatial econometric model. From 2000 to 2020, there were significant spatial spillover effects in the economic growth of 31 provinces in China. The flow of capital and labor factors between provinces exerted adverse or inhibitory effects on the economic development of neighboring regions. In contrast, the input of land factors influenced the economic development of adjacent areas through specific spatial spillover effects.
3. The changes in the contribution rates of production factors in various provinces from 2000 to 2020 are consistent with the national trend. The contribution rate of the capital factors exhibits significant regional differences with a notable "east-low, west-high" pattern, decreasing year by year. The reliance on capital factors is relatively low in the Yangtze River Economic Belt and the southeastern coastal areas. The contribution rate of the labor factors decreases yearly, although provinces with large populations still rely on the labor factors. The contribution rate of land factor decreases yearly in most provinces, indicating a reduced dependency on land. The contribution rate of the technology factor shows a significant rise, with higher rates observed in economically developed areas along the southeastern coast and the Yangtze River Economic Belt. Analyzing different production factor contribution rates can identify each province’s high-quality development progress.

### Policy recommendations

China has undergone different development stages since the reform and opening up. This study profoundly explores the stage characteristics of China’s economic growth from the perspective of the contribution rate of production factors and the spatial characteristics from the provincial perspective and draws a series of valuable conclusions. It was found that we have formed a profound understanding of the path and characteristics of China’s high-quality development. Based on this, this article further puts forward the following policy suggestions:

1. Strengthen the core position of scientific and technological innovation in high-quality development. Research has found that scientific and technological innovation contributes significantly to the high-quality development of China’s economy, so it is necessary to continue to play an essential role in scientific and technological innovation. The Chinese government can increase investment in scientific research, development, and innovation, support further improvement of the efficiency of transformation of scientific and technological achievements, promote the industrialization of scientific and technological achievements, and ultimately enhance the main force of scientific and technological innovation.
2. Improve resource utilization efficiency and environmental protection levels. The study found that China’s resource utilization efficiency has been significantly improved during the high-quality development stage, but it still needs to improve its capabilities in this area in the face of global sustainable development goals. This requires adhering to the goal of green and low-carbon development, effectively reducing carbon emissions, accelerating the development of clean energy and energy-saving technologies, exploring carbon-neutral technologies, and ensuring efficient use of resources and adequate environmental protection.
3. Promote the reasonable flow of resource elements between regions. From the perspective of spatial characteristics research, significant regional differences exist in the high-quality development of China’s economy. Considering that excessive outflow of mobile factors such as capital and labor in economically underdeveloped areas will have a negative impact on economic growth. In this regard, the Chinese government should further clarify the main functions of the regions, deeply explore the ecological compensation mechanism, give full play to the resource endowments and comparative advantages of each region through regional cooperation, and promote the gradient transfer of industries along the life cycle stage to balance the development gap between regions.

## Supporting information

S1 File Appendix. (DOC)

S2 File Raw data (China National Data and China Province Data). (XLSX)
