# Supplementary material for: Classification and spatial characteristics of different development stages in China: Evidence from the contribution rate of production factors
Source: PLoS One. 2025 Jan 14;20(1):e0313069. doi: 10.1371/journal.pone.0313069 (PMC11981536; doi:10.1371/journal.pone.0313069)
Supplement: S1 File — (DOC) [file pone.0313069.s001.doc]

**Table 1. Contribution rate of production factors in each province from 2000 to 2020**

| province | 2007 | | | | 2012 | | | | 2020 | | | |
| --- | --- | --- | --- | --- | --- | --- | --- | --- | --- | --- | --- | --- |
|  | K | L | S | A | K | L | S | A | K | L | S | A |
| Beijing | 26.93% | 27.14% | 25.90% | 20.03% | 23.91% | 25.16% | 20.48% | 30.45% | 19.77% | 19.30% | 17.77% | 43.17% |
| Tianjin | 31.98% | 28.24% | 28.45% | 11.33% | 29.75% | 21.81% | 22.55% | 25.89% | 25.67% | 20.61% | 20.92% | 32.80% |
| Hebei | 31.07% | 27.69% | 24.37% | 16.87% | 29.69% | 25.97% | 23.18% | 21.16% | 27.73% | 23.84% | 21.71% | 26.72% |
| Shanxi | 31.55% | 30.91% | 22.39% | 15.14% | 29.50% | 28.75% | 21.97% | 19.78% | 26.93% | 26.00% | 20.98% | 26.08% |
| Inner Mongolia | 34.57% | 28.80% | 24.40% | 12.23% | 30.93% | 25.20% | 23.83% | 20.05% | 31.03% | 20.25% | 19.85% | 28.87% |
| Liaoning | 31.96% | 29.40% | 24.38% | 14.27% | 30.16% | 26.04% | 22.57% | 21.24% | 30.13% | 24.10% | 21.44% | 24.33% |
| Jilin | 36.13% | 28.88% | 23.00% | 11.99% | 34.09% | 23.35% | 23.47% | 19.09% | 35.81% | 20.41% | 21.81% | 21.97% |
| Heilongjiang | 30.05% | 28.13% | 29.54% | 12.28% | 31.15% | 26.07% | 25.28% | 17.50% | 32.43% | 25.07% | 20.87% | 21.63% |
| Shanghai | 26.34% | 26.04% | 26.87% | 20.74% | 23.62% | 20.41% | 25.62% | 30.34% | 19.19% | 18.00% | 18.75% | 44.07% |
| Jiangsu | 27.20% | 27.17% | 23.62% | 22.01% | 24.27% | 23.04% | 21.16% | 31.53% | 18.82% | 19.51% | 19.06% | 42.61% |
| Zhejiang | 28.30% | 25.78% | 25.18% | 20.74% | 24.56% | 23.01% | 20.53% | 31.91% | 19.28% | 17.99% | 18.44% | 44.28% |
| Anhui | 28.72% | 31.50% | 25.75% | 14.02% | 23.48% | 26.74% | 26.47% | 23.31% | 22.34% | 21.22% | 20.22% | 36.22% |
| Fujian | 29.40% | 27.98% | 25.40% | 17.22% | 25.63% | 25.18% | 20.66% | 28.53% | 24.37% | 21.55% | 19.68% | 34.40% |
| Jiangxi | 28.41% | 28.55% | 26.22% | 16.81% | 27.36% | 25.35% | 24.13% | 23.17% | 26.80% | 23.66% | 21.47% | 28.07% |
| Shandong | 28.24% | 33.12% | 25.54% | 13.10% | 26.89% | 30.67% | 24.13% | 18.31% | 25.67% | 27.34% | 20.81% | 26.17% |
| Henan | 28.42% | 31.00% | 24.10% | 16.48% | 26.89% | 28.07% | 22.29% | 22.75% | 25.78% | 26.98% | 21.21% | 26.04% |
| hubei | 29.75% | 28.38% | 25.71% | 16.15% | 23.59% | 24.47% | 22.56% | 29.38% | 22.07% | 22.19% | 22.15% | 33.58% |
| Hunan | 29.34% | 28.77% | 25.87% | 16.02% | 28.60% | 21.78% | 20.07% | 29.56% | 25.21% | 21.73% | 19.09% | 33.97% |
| Guangdong | 28.53% | 26.35% | 24.85% | 20.26% | 20.92% | 25.61% | 21.95% | 31.52% | 19.32% | 23.85% | 18.98% | 37.85% |
| Guangxi | 30.52% | 30.65% | 28.61% | 10.21% | 31.54% | 28.35% | 23.53% | 16.58% | 35.96% | 26.95% | 23.15% | 13.94% |
| Hainan | 29.08% | 30.26% | 25.58% | 15.08% | 28.26% | 27.35% | 23.29% | 21.11% | 30.43% | 21.28% | 20.67% | 27.62% |
| Chongqing | 29.41% | 29.88% | 27.57% | 13.14% | 24.11% | 25.61% | 23.85% | 26.43% | 22.19% | 20.34% | 22.50% | 34.97% |
| Sichuan | 29.98% | 28.76% | 25.42% | 15.84% | 28.86% | 25.64% | 22.17% | 23.34% | 26.23% | 22.61% | 23.48% | 27.68% |
| Guizhou | 33.11% | 31.92% | 24.19% | 10.79% | 35.15% | 28.81% | 21.99% | 14.05% | 38.81% | 23.69% | 20.75% | 16.76% |
| Yunnan | 31.73% | 26.93% | 27.69% | 13.65% | 35.28% | 25.25% | 25.06% | 14.41% | 36.21% | 24.06% | 20.91% | 18.82% |
| Xizang | 38.24% | 27.20% | 30.39% | 4.18% | 37.31% | 25.97% | 22.49% | 14.23% | 37.00% | 22.40% | 22.49% | 18.11% |
| Shaanxi | 30.72% | 29.32% | 25.33% | 14.64% | 32.49% | 27.62% | 22.19% | 17.71% | 31.02% | 26.87% | 19.33% | 22.79% |
| Gansu | 32.43% | 30.32% | 21.89% | 15.37% | 35.09% | 27.94% | 20.08% | 16.89% | 35.55% | 22.45% | 23.47% | 18.53% |
| Qinghai | 40.50% | 28.70% | 21.98% | 8.83% | 36.07% | 25.63% | 21.60% | 16.70% | 38.26% | 22.67% | 20.30% | 18.78% |
| Ningxia | 37.56% | 29.65% | 21.02% | 11.77% | 35.76% | 27.91% | 22.15% | 14.18% | 37.39% | 23.62% | 21.25% | 17.75% |
| Xinjiang | 36.17% | 30.40% | 25.68% | 7.74% | 34.21% | 26.95% | 26.23% | 12.61% | 35.00% | 24.54% | 25.22% | 15.24% |
